# Development of a Nongenetic Mouse Model of Type 2 Diabetes

**DOI:** 10.1155/2011/416254

**Published:** 2011-11-29

**Authors:** Elizabeth R. Gilbert, Zhuo Fu, Dongmin Liu

**Affiliations:** Department of Human Nutrition, Foods and Exercise, College of Agriculture and Life Sciences, Virginia Tech, Blacksburg, VA 24061, USA

## Abstract

Insulin resistance and loss of *β*-cell mass cause Type 2 diabetes (T2D). The objective of this study was to generate a nongenetic mouse model of T2D. Ninety-six 6-month-old C57BL/6N males were assigned to 1 of 12 groups including (1) low-fat diet (LFD; low-fat control; LFC), (2) LFD with 1 i.p. 40 mg/kg BW streptozotocin (STZ) injection, (3), (4), (5), (6) LFD with 2, 3, 4, or 5 STZ injections on consecutive days, respectively, (7) high-fat diet (HFD), (8) HFD with 1 STZ injection, (9), (10), (11), (12) HFD with 2, 3, 4, or 5 STZ injections on consecutive days, respectively. After 4 weeks, serum insulin levels were reduced in HFD mice administered at least 2 STZ injections as compared with HFC. Glucose tolerance was impaired in mice that consumed HFD and received 2, 3, or 4 injections of STZ. Insulin sensitivity in HFD mice was lower than that of LFD mice, regardless of STZ treatment. Islet mass was not affected by diet but was reduced by 50% in mice that received 3 STZ injections. The combination of HFD and three 40 mg/kg STZ injections induced a model with metabolic characteristics of T2D, including peripheral insulin resistance and reduced *β*-cell mass.

## 1. Introduction

It is estimated that 23.6 million or 8% of the American population suffers from diabetes [1]. Almost the same numbers have prediabetes. While the availability of novel drugs, techniques, and surgical intervention has improved the survival rate of individuals with diabetes, the prevalence of diabetes still rises in Americans, and the number of people with diabetes is projected to double by 2025 [2]. These alarming statistics underscore the need for research aimed at discovering novel therapeutic strategies for the prevention or treatment of this disease. For the study of Type 2 diabetes (T2D) there are a variety of inbred mouse strains that serve as popular models [3]. Genetic obese diabetic mice, including leptin and leptin receptor knockouts (ob/ob and db/db, resp.), are commonly used T2D models for developing new treatments for this disease [3]. However, T2D is a disease stemming from a combination of cumulative polygenic traits and interactions with environmental stressors [4]. A diet-induced model can be used to more closely resemble the gradual progression from obesity to insulin resistance and T2D, the hallmarks of the human phenotype [4]. The development of a nongenetic mouse model for T2D involves establishment of insulin resistance, a period of compensatory insulin release to maintain glucose homeostasis. The pathogenesis of T2D then involves a combination of factors including destruction of *β* cells that reduces insulin secretion, hindering the body's ability to maintain normoglycemia. Because T2D is such a complex, heterogeneous disease resulting from a combination of genetic and lifestyle-induced factors, there is unlikely to be a single model that best fits all T2D scenarios [5].

There have been multiple studies described that involve the development of a C57BL/6 model of T2D [6]. Luo et al. [6] started their study with 3-week-old mice and administered a single injection of STZ at 100 mg/kg BW after 3 wk of feeding a high-fructose, high-fat, or standard chow diet to induce diabetes. T2D often occurs in middle- to older-aged individuals. Therefore, a mouse model that closely represents the physiological state preceding insulin resistance and T2D may be more suitable for mimicking this human disease. In addition, insulin resistance and hyperinsulinemia manifested after the onset of obesity represent a fundamental aspect of T2D etiology [7]. Luo et al. [6] described two criteria for a suitable model of T2D: (1) metabolic profile matching the disease and (2) cost-effectiveness. Several groups [8, 9] used adult rats and several weeks of providing a high-fat diet followed by a single, relatively high dose of STZ injection to induce a model of T2D. In a recent publication we demonstrated that five consecutive low doses (40 mg/kg BW) of STZ triggered mild to moderate diabetes in young mice fed standard rodent chow by causing gradual destruction of pancreatic *β* cells [10]. Given this observation and our hypothesis that pancreatic *β* cells in obese mice are more susceptible to environmental factor-induced injury, we developed a method to generate a nongenetic model of T2D using older obese mice. We employed a combination of feeding a high-fat diet and administering consecutive multiple low doses of STZ injection, without causing diabetes in standard chow-fed mice. This protocol results in a gradual, partial destruction of pancreatic *β* cells in high fat-fed, insulin-resistant mice without significant effect on islets in chow-fed mice, more closely resembling the features of T2D in humans.

## 2. Materials and Methods

### 2.1. Animals and Study Design

The following animal procedures were reviewed and approved by the institutional animal care and use committee at Virginia Polytechnic Institute and State University. All chemicals were purchased from Sigma Aldrich (St. Louis, MO) unless otherwise stated. Diets were purchased from Research Diets Inc. (New Brunswick, NJ). Male C57BL/6NCr (NCI Frederick) mice (6 mo. old) were obtained and housed individually in standard-sized cages (29 × 14 × 13 cm) arranged in a double-faced 140-cage ventilation rack in a temperature and humidity controlled, pathogen-free room on a 12 h light cycle (6 am to 6 pm) with free access to food and water. A randomized complete block design was used to assign 96 mice to one of 12 groups with fasting blood glucose (FBG) as the blocking factor. Mean FBG and body weight (BW) after assignment to groups was 114.38 mg/dL and 28.08 g, respectively. Body composition was evaluated before the start of the trial using an LF-90 instrument (Bruker Optics, Inc., Billerica MA). The LF-90 body composition instrument is based on Time Domain Nuclear Magnetic Resonance (TD-NMR) technology which provides an in vivo measurement of lean tissue, body fat, and body fluid in live mice and rats without anesthesia. Mean lean, fat and fluid percentages were similar among groups (means of 72.74 ± 2.2, 13.36 ± 2.8, and 7.37 ± 0.38%, resp.). Mice were offered either a standard rodent chow containing 10% of kcal as fat (low-fat diet; LFD) or a diet containing 60% kcal as fat, primarily in the form of saturated fat from lard (high-fat diet; HFD; Table 1) ad libitum. Mice received an intraperitoneal (i.p.) injection of vehicle (0.05 M citrate buffer, pH 4.5; non-STZ LFD and HFD controls) or 1, 2, 3, 4, or 5 doses of STZ dissolved in the vehicle at 40 mg/kg BW per dose. Streptozotocin was prepared fresh each day and multiple doses were administered on consecutive days. At 1 wk following the first injection, FBG levels were measured. At 2 and 3 wk following the first injection, a glucose tolerance test (GTT) and insulin tolerance test (ITT) were performed, respectively. At 4 wk following STZ injections, FBG was measured, as well as body composition, and animals were euthanized by CO_2_ asphyxiation after an overnight fast. Blood was collected from the orbital sinus into heparin-coated capillary tubes to prevent clotting, and the remainder was collected in microcentrifuge tubes and allowed to clot on ice. The intra-abdominal fat pad, liver, and pancreas were surgically removed from the mouse, rinsed in ice-cold PBS and weighed. The pancreas was divided into the “head” portion (adjacent to the duodenum) and the remainder (“body”) was divided into 3 equal portions. Tissues were fixed in 10% neutral-buffered formalin. Pancreas samples were sent to AML Laboratories (Rosedale, MD) for paraffin-embedding, sectioning, and mounting on glass slides. Three sections were made for each tissue sample, such that there were 12 total sections per pancreas. Slides were stained with hematoxylin and eosin and used for measurement of islet mass

### 2.2. Blood Lipid and Hormone Profile Parameters 

Total cholesterol was immediately measured from total blood following euthanasia using a CardioChek blood analyzer (Polymer Technology Systems, Indianapolis, IN; PTS). Clotted blood samples were centrifuged at top speed for 10 min. The supernatant was transferred to a new tube and centrifuged at top speed for an additional 5 min. The supernatant was collected and transferred to a 96-well plate for storage at −80°C. Serum was used for measuring insulin using an ultrasensitive mouse/rat insulin ELISA (Mercodia, Inc., Uppsala, Sweden). Supernatants from centrifuged blood collected with heparinized capillaries (plasma) used for measuring triglycerides and HDL with the CardioCheck analyzer and appropriate test strips. Randomly selected plasma samples were used to determine cholesterol levels and to recalculate HDL and triglycerides on a whole blood basis. The HDL levels were calculated using the Friedewald equation: [LDL-Cholesterol = Total Cholesterol–(HDL-Cholesterol + Triglycerides/5)], with all concentrations presented as mg/dL.

### 2.3. Fasting Blood Glucose, Glucose Tolerance Test (GTT), and Insulin Tolerance Test (ITT)

At 2, 4, 6, 7, 8, and 9 wk, 16-h FBG levels were measured in whole blood drawn from the tail vein using an ultrasensitive hand-held glucometer (The Kroger Co., Cincinnati, OH). Both the glucose and insulin tolerance tests were performed following a 16 h fast with baseline blood glucose measurements taken immediately before beginning the test. Blood glucose measurements were taken by a single person to eliminate potential operator variability. For the GTT, glucose was delivered via i.p. injection at a dose of 2 g · kg^−1^  BW in a volume of 0.1 mL water. Blood glucose levels were measured at 15, 30, 60, and 120 min after injection. For the ITT, human insulin was administered via i.p. injection at a dose of 0.75 U · kg^−1^  BW. Blood glucose levels were measured at 15, 30, 60, and 120 min after injection as described for GTT test.

### 2.4. Islet Mass and Density

Histological evaluation was performed in 4 groups of mice: (1) control mice that consumed LFD, (2) mice that consumed LFD and received 3 injections of STZ, (3) control mice that consumed HFD, and (4) mice that consumed HFD and received 3 injections of STZ. Islet mass was determined using point counting stereology [11] on hematoxylin and eosin-stained slides. A 100-square grid reticle (0.1 mm per square) was used to count points over islet tissue using an Olympus BX51 microscope and a 10x magnification. The area occupied by islets was expressed as a ratio to the total area of pancreatic tissue on the slide and multiplied by 100 to determine relative islet volume percentage. Islet mass was calculated as the relative volume of islets multiplied by the total weight of the pancreas. Images of all sections were acquired with a CCD camera and analyzed using Image J software to determine pancreas area on the slide. The area (mm^2^) values were generated automatically. Relative islet volume was analyzed to compare the head region to the body of the pancreas and to evaluate total volume of the entire pancreas tissue. 

### 2.5. Statistical Analysis

The Proc Univariate procedure of SAS was used to test for normality, and data were transformed to normalize variances using a transformation selector as previously described [12]. All data were analyzed using the Proc Mixed procedure of SAS (Cary, NC). An alpha level of *P* < 0.05 was considered statistically significant. For the glucose tolerance and insulin tolerance tests, incremental area under the curve and total area under the curve (AUC) was calculated. Incremental AUC were calculated as the sum of blood glucose measurements for two consecutive time points multiplied by the time interval and then divided by two (trapezoidal rule). The glucose clearance rate was also calculated for each animal from the glucose tolerance test data by determining the slope of the line (*m* = (*y* ^1^ − *y* ^2^) · (*x* ^1^−*x* ^2^)^−1^) for blood glucose measurements between 30 and 120 min. This calculation was based on the assumption that the blood glucose levels of “normal” lean mice will peak at 30 min post-glucose loading, thus, clearance rates can be determined from the slope of the line between 30 and 120 min [5]. The model for the GTT AUC, ITT AUC, body composition before and after STZ, and blood hormone and lipid profile parameters collected on the day of euthanasia included the main effects of diet and STZ treatment and the 2-way interaction. Orthogonal linear contrasts were used to further test for significant differences. Contrast coefficients were generated to compare the control to the treated within each diet and to compare each low-fat group with its respective high-fat group. Mouse nested within diet × STZ treatment was designated in the model as a random effect and used as the error term. The raw GTT and ITT data, as well as the fasting blood glucose, BW and feed intake data that were collected longitudinally, were summarized as least squares means with SEM plotted over time, and the statistical model included the main effects of diet, STZ treatment and time, and 2-way interactions, with time as a repeated measure. For each variable analyzed, mouse nested within diet × STZ treatment was subjected to at least 3 covariance structures including autoregressive order 1, unstructured covariance and compound symmetry. For all variables, the Akaike's information criterion was closest to zero using the autoregressive order 1 covariance structures, and thus, it was used for each analysis with the Kenward-Roger adjustment method for approximation of degrees of freedom. The model for relative islet volume also included pancreas region (head versus body) as a main effect.

## 3. Results

### 3.1. Food Intake, Body and Organ Weight, and Body Composition

**Figure 1:**
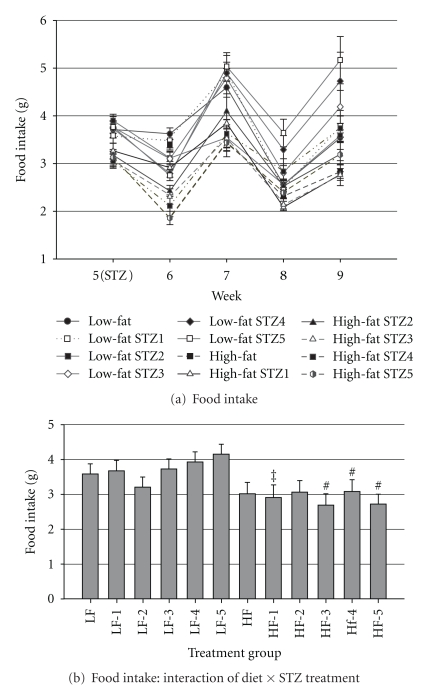
Food intake. (a) Food intake (A; g · mouse^−1^ · d^−1^) before beginning STZ injections (end of wk 5) and each week until the end of the trial (9 wks) and (b) interaction of diet × STZ treatment on food intake. Male, retired breeder (6 mo.) C57BL/6 mice were used for this study. Before STZ injections *N* = 8 and after STZ injections *N* = 5–8. Values represent mean ± SEM. ^#^ *P* < 0.05 versus respective LFD group, ^‡^ *P* = 0.07 versus respective LFD group.

Feed intake data are shown in Figure 1. During the first week following STZ injections, feed intake was reduced in mice that consumed the HFD as compared with the noninjected mice that consumed HFD (*P* < 0.01; Figure 1(a)). After the first week post-STZ, food intake was not different among the STZ-injected and non-STZ injected mice, regardless of diet. Mice that consumed HFD and received 3, 4, or 5 injections of STZ ate significantly less (*P* < 0.01) than the respective LFD groups (Figure 1(b)). 

**Figure 2:**
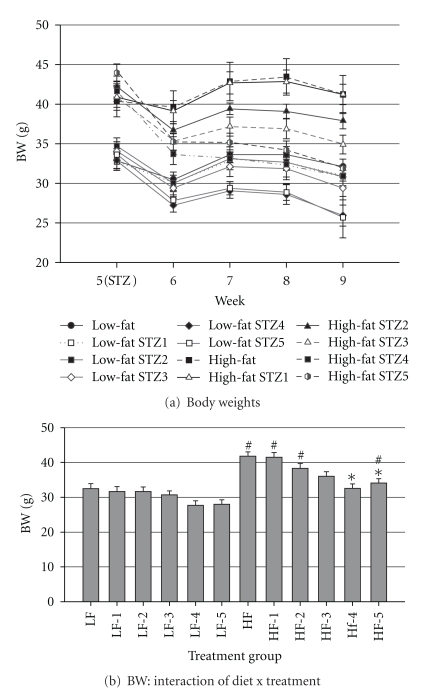
Body weights: (a) Body weights before beginning STZ injections (end of wk 5) and each week until the end of the trial (9 wks) and (b) interaction of diet and STZ treatment on BW. Male, retired breeder (6 mo.) C57BL/6 mice were used for this study. Before STZ injections *N* = 8 and after STZ injections *N* = 5–8. Values represent mean ± SEM. *P < 0.05 versus respective control, ^#^ *P* < 0.05 versus respective LFD group.

After 5 wk of ad libitum feeding but before STZ injections, mice that consumed the HFD were heavier than mice that consumed the LFD (41.7 ± 3.8 versus 33.5 g ± 2.8, resp.). During the first week that followed STZ injections, BW decreased in response to STZ injections and the decrease was most accentuated in mice that received the most injections of STZ (Figure 2(a)). Injection of STZ had no effect on BW in mice that consumed the LFD, whereas mice that consumed HFD and received 4 or 5 injections of STZ weighed less (*P* < 0.04) than the respective control mice. Mice that consumed HFD and received 1, 2, or 5 injections of STZ weighed more than their LFD-fed counterparts (*P* < 0.04), while there was no significant difference among LFD and HFD mice that received 3 or 4 injections. 

**Figure 3:**
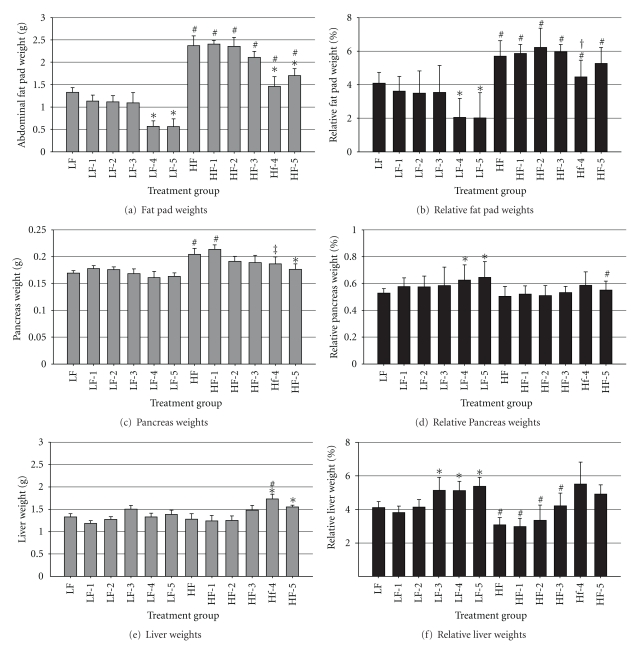
Organ weights: (a) Intra-abdominal fat pad weights as absolute values and (b) as percentages of BW on final day of trial. (c) Pancreas weight as absolute value and (d) as percentage of BW on final day of trial. (e) Liver weight as absolute value and (f) as percentage of BW on final day of trial. Male, retired breeder (6 mo.) C57BL/6 mice were used for this study. Values represent mean ± SEM (*N* = 5–8). *P < 0.05 versus respective control, ^#^ *P* < 0.05 versus respective LFD group, ^‡^ *P* = 0.07 versus respective LFD group, ^†^ *P* = 0.05 versus respective control.

The abdominal fat pad, both as an absolute weight and as a percentage of BW, was different among groups (*P* = 0.0001), and specifically, was greater (*P* < 0.05) in mice that consumed HFD, regardless of STZ treatment, compared with mice that consumed LFD (Figures 3(a) and 3(b)). In mice that consumed LFD, 4 or 5 STZ injections reduced the fat pad weight, both as an absolute weight and as percentage of BW (*P* < 0.05). In mice that consumed HFD, 4 or 5 STZ injections reduced absolute fat pad weight but had no effect on relative fat pad weight. The pancreas weights and relative pancreas weights were different among treatment groups as well (*P* = 0.002 and *P* = 0.04, resp.). The weight of the pancreas was not different among mice that consumed LFD (Figure 3(c)), but when expressed relative to BW was increased in mice that received 4 or 5 injections of STZ (Figure 3(d)). The pancreas weight was greater in mice that consumed HFD and received 1 injection of either vehicle or STZ, as compared with the respective LFD-fed group (*P* < 0.05). Mice that consumed HFD and received 5 injections of STZ exhibited reduced pancreas weights as compared with HFD controls (*P* < 0.05) and when expressed relative to BW showed reduced weights as compared with the LFD group that received 5 injections of STZ (*P* < 0.05). The liver weights and relative liver weights were also different among treatment groups (*P* = 0.003 and *P* = 0.0001 for interaction of diet × STZ treatment, resp.). The weight of the liver was not affected by STZ in mice that consumed LFD (Figure 3(e)) but when expressed proportionally to BW was greater (*P* < 0.05) in mice that received 3, 4, or 5 injections of STZ (Figure 3(f)). In mice that consumed the HFD and received 4 or 5 injections of STZ, the absolute weight of the liver was greater (*P* < 0.05) as compared with the HFD controls, however, when expressed as a ratio to BW, there were no differences among mice that consumed HFD. Mice that consumed HFD and received 4 injections of STZ had greater (*P* < 0.05) liver weights as compared with the respective LFD-fed group, and when liver was expressed relative to BW, the liver was bigger in mice that consumed LFD and either received the vehicle or up to 3 injections of STZ, as compared with the respective group of mice that consumed HFD. 

**Figure 4:**
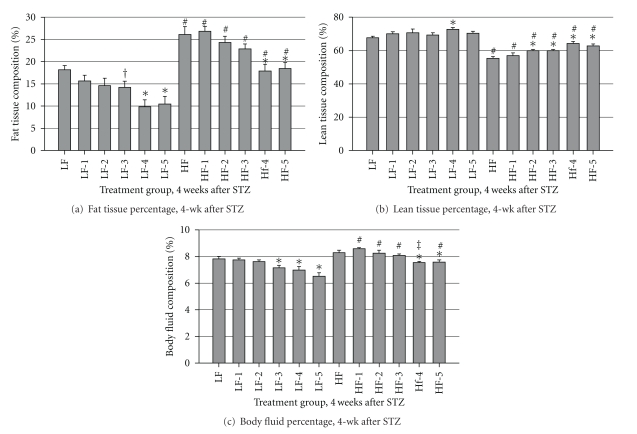
Body composition after STZ: (a) Fat tissue percentage, (b) lean tissue percentage, and (c) fluid percentage of body composition during the final week of trial. Male, retired breeder (6 mo.) C57BL/6 mice were used for this study. Values represent mean ± SEM (*N* = 5–8). *P < 0.05 versus respective control, ^#^ *P* < 0.05 versus respective LFD group, ^‡^ *P* = 0.06 versus respective LFD group, ^†^ *P* = 0.07 versus respective control.

Body composition reflected feed intake and BW data and was significantly altered by STZ treatment. After 1 mo. of feeding, the mean fat tissue, lean tissue, and body fluid percentages for the mice that consumed HFD and LFD were 23.7 ± 0.74 and 14.01 ± 0.77, 60.58 ± 0.67 and 70.24 ± 0.91, and 8.56 ± 0.08 and 7.57 ± 0.15, respectively. After 4 wk of STZ treatment, fat tissue percentage was reduced (*P* < 0.05) in mice that were administered 4 or 5 injections of STZ and consumed either the LFD or HFD, in comparison to the control animals (Figure 4(a)). Lean percentage increased in mice that consumed the LFD and were administered 4 injections of STZ (Figure 4(b)). In mice that consumed HFD, the effect was more pronounced and mice that received 2, 3, 4, or 5 injections of STZ displayed increased (*P* < 0.05) lean tissue percentages. Fluid percentage decreased (*P* < 0.05) in mice that consumed LFD and received at least 3 injections of STZ, in comparison to the controls, and in mice that consumed HFD and received at least 4 injections of STZ (Figure 4(c)). 

### 3.2. Fasting Blood Glucose

**Figure 5:**
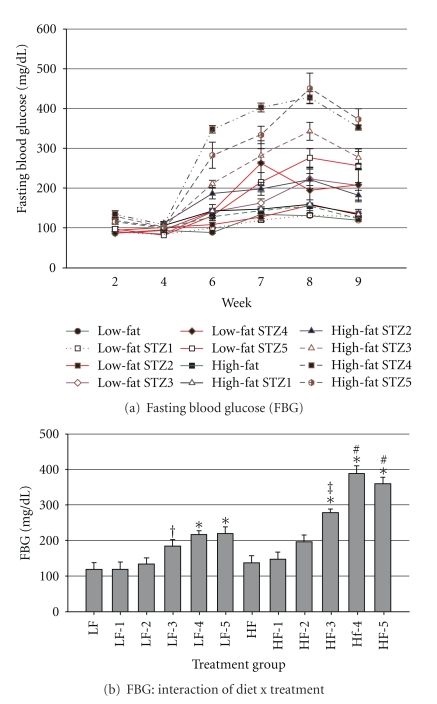
Fasting blood glucose concentrations: (a) Fasting blood glucose concentration before beginning STZ (end of wk 2) and each week until the end of the trial (9 wks) and (b) interaction of diet and STZ treatment on fasting blood glucose concentrations. Male, retired breeder (6 mo.) C57BL/6 mice were used for this study. Values represent mean ± SEM (*N* = 5–8). *P < 0.05 versus respective control, ^#^ *P* < 0.05 versus respective LFD group, ^‡^ *P* = 0.08 versus respective LFD group, ^†^ *P* = 0.08 versus respective control.

Fasting blood glucose levels were measured after 2, 4, 6, 7, 8, and 9 wk of the study (Figure 5(a)). After 4 wk of feeding and before injection of STZ, fasting blood glucose levels were not significantly different between LFD- and HFD-fed mice (89.2 ± 19.7 versus 104.1 ± 20.0 mg/dL, resp.). In response to STZ,however, the differences in fasting blood glucose levels became accentuated (*P* = 0.0001) and mice that consumed the LFD were more resistant to the effects of STZ. In the first wk following the start of STZ injections, only mice that received at least 3 injections of STZ and consumed HFD displayed fasting blood glucose levels that were significantly different from the HFD controls (*P* < 0.01; Figure 5(b)). Mice that received at least 3 injections of STZ and consumed the HFD also had greater fasting blood glucose levels than the respective LFD group (*P* < 0.05). Over the 4 wk period that followed STZ injections, mice that consumed LFD and received 4 or 5 injections of STZ displayed fasting blood glucose levels that were different from the LFD-fed control (*P* < 0.05). 

### 3.3. Glucose Tolerance

**Figure 6:**
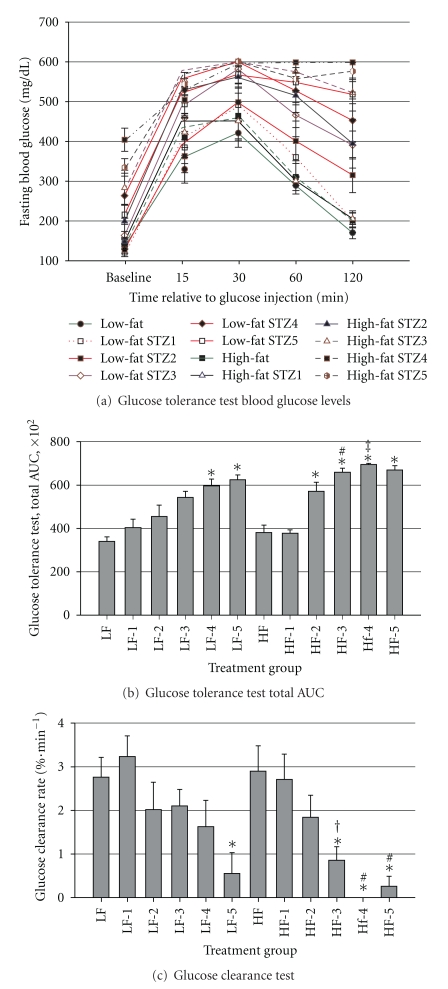
Glucose tolerance test: (a) Fasting blood glucose concentrations at baseline and 15, 30, 60, and 120 min post-glucose loading via i.p. injection (2 g · kg^−1^  BW), (b) interaction of diet and STZ treatment on total AUC calculated from glucose tolerance test data, (c) interaction of diet and STZ treatment on glucose clearance rate, calculated from 30 to 120 min post-glucose loading. Male, retired breeder (6 mo.) C57BL/6 mice were used for this study. Values represent mean ± SEM (*N* = 5–8). *P < 0.05 versus respective control, ^#^ *P* < 0.05 versus respective LFD group, ^‡^ *P* = 0.06 versus respective LFD group, ^†^ *P* = 0.06 versus respective control.

The glucose tolerance test data were analyzed as total AUC and glucose clearance rate between 30 and 120 min. Means and standard errors plotted over time are shown in Figure 6(a), and AUC and glucose clearance rate data are illustrated in Figures 6(b) and 6(c), respectively. There was a greater AUC for mice that consumed either the LFD or HFD and received 4,5 or 2, 3, 4, or 5 injections, respectively, of STZ as compared with the respective control mice (*P* < 0.05). The AUC was greater in mice that consumed HFD and received 3 (*P* < 0.05) or 4 (*P* = 0.06) injections of STZ as compared to the respective LFD group. The glucose clearance rate was substantially reduced by STZ treatment (*P* = 0.0001; Figure 6(c)). In mice that ate the LFD and received 5 injections of STZ, glucose clearance rate was reduced (*P* = 0.001). The HFD magnified the effects of STZ on glucose clearance. In mice that consumed the HFD, 3 low doses of STZ is sufficient to significantly impair glucose clearance rate (*P* < 0.01), but 5 injections of STZ is required to reduce glucose clearance rate (*P* = 0.02) in mice that consumed LFD.

### 3.4. Insulin Tolerance

**Figure 7:**
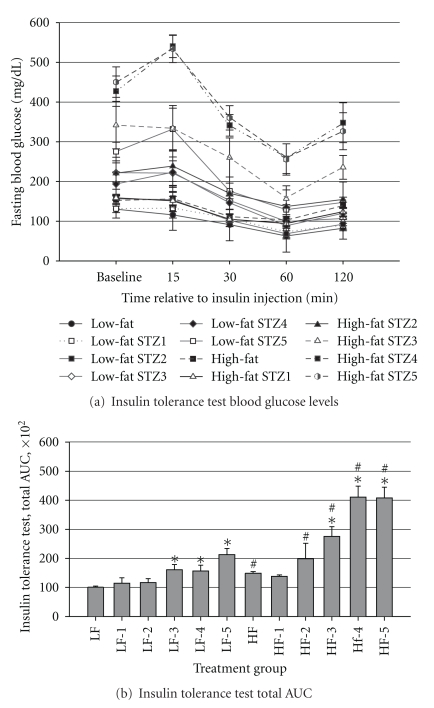
Insulin tolerance test: (a) Fasting blood glucose concentrations at baseline and 15, 30, 60, and 120 min post-insulin loading via i.p. injection (0.75 U · kg^−1^  BW), (b) interactions of diet × STZ treatment on total AUC calculated from insulin tolerance test data. Male, retired breeder (6 mo.) C57BL/6 mice were used for this study. Values represent mean ± SEM (*N* = 5–8). ^#^ *P* < 0.05 versus respective LFD group.

The insulin tolerance test data were also analyzed as total AUC. Means and standard errors plotted over time are shown in Figure 7(a) and total AUC in Figure 7(b). The AUC was affected by diet and STZ treatment (*P* = 0.0001). Mice fed HFD had significantly higher AUC that those treated with LFD (*P* < 0.05). There was a greater AUC in mice that consumed either the LFD or HFD and received at least 3 injections of STZ, as compared with the respective control group (*P* < 0.05), suggesting that STZ treatment may partially cause insulin intolerance in these animals. With the exception of mice that received 1 injection of STZ, the total AUC for all groups of mice that consumed HFD was greater (*P* < 0.05) than the AUC for the respective groups of mice that ate the LFD. These data indicate that HFD treatment for 4 wks can adequately induce insulin resistance in older adult mice.

### 3.5. Blood Lipid Profile and Hormone Parameters

**Figure 8:**
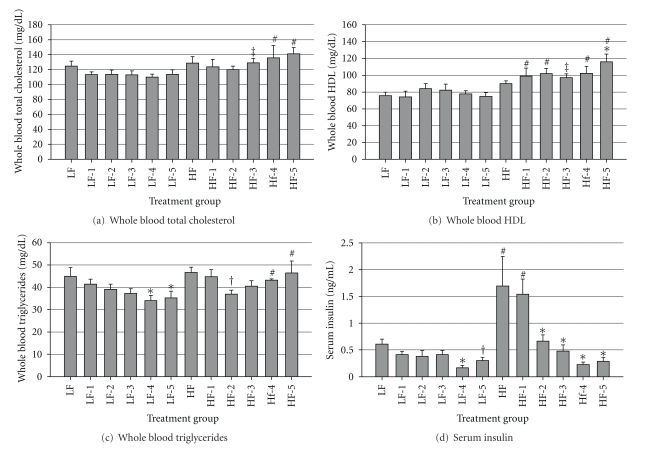
Interactions of diet × STZ treatment on blood lipid profile and hormones on final day of trial. (a) Whole blood total cholesterol, (b) whole blood HDL, (c) whole blood triglycerides, and (d) serum insulin levels. Male, retired breeder (6 mo.) C57BL/6 mice were used for this study. Values represent mean ± SEM (*N* = 5–8). *P < 0.05 versus respective control, ^#^ *P* < 0.05 versus respective LFD group, ^‡^ *P* = 0.07 to 0.08 versus respective LFD group, ^†^ *P* = 0.06 to 0.07 versus respective control.

At 4 wk after STZ, total blood cholesterol levels were greater (*P* < 0.05) in mice that consumed the HFD and received 4 or 5 injections of STZ as compared with the respective LFD groups (Figure 8(a)). There tended (*P* = 0.07) to be greater cholesterol levels in mice that consumed HFD and received 3 injections as compared with the respective LFD group. The total blood HDL levels were greater (*P* < 0.05) in mice that consumed HFD and received 5 STZ injections as compared with the HFD controls (Figure 8(b)). Mice that consumed HFD and received 1, 2, 4, or 5 injections of STZ displayed greater (*P* < 0.05) HDL concentrations than the respective LFD groups, and mice that consumed HFD and received 3 injections of STZ tended (*P* = 0.08) to have greater HDL blood concentrations than the respective LFD group. Mice that ate the HFD and received 4 or 5 injections of STZ displayed greater (*P* < 0.03) blood triglyceride levels in the blood as compared with the respective mice that were fed the LFD diet. Total blood triglyceride levels were reduced (*P* < 0.04) in mice that consumed LFD and received 4 or 5 doses of STZ, as compared with the controls (Figure 8(c)).

Consistent with developing insulin resistance in HFD-fed mice, the blood insulin levels in HFD-fed mice were about threefold of those in LFD control mice (*P* = 0.001), suggesting that treatment of older adult mice with HFD for 4 wks can induce hyperinsulinemia, a metabolic feature during the pathogenesis of T2D. In HFD mice, at least 2 injections of STZ were needed to reduce serum insulin levels (*P* = 0.001) as compared with HFD controls, whereas 4 consecutive injections of STZ were required to cause significant reductions in circulating insulin (*P* = 0.006) in mice that consumed the LFD (Figure 8(d)). 

### 3.6. Relative Islet Volume and Total Islet Mass

**Figure 9:**
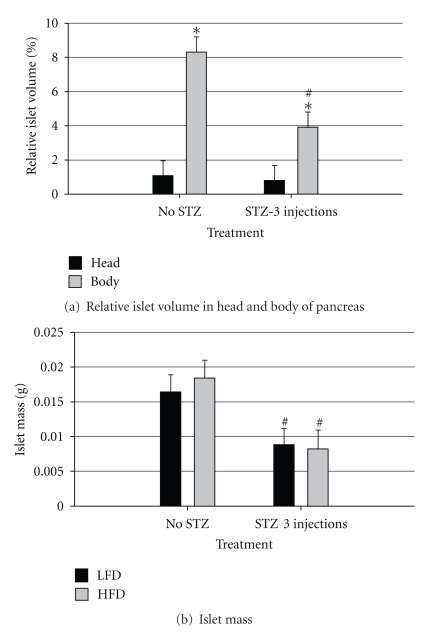
Relative islet volume and total islet mass on the final day of the trial. (a) Relative islet volume and (b) total islet mass. Male, retired breeder (6 mo.) C57BL/6 mice were used for this study. Values represent mean ± SEM (*N* = 5–8). *P = 0.0001, versus pancreas head region in mice receiving the same treatment (interaction of pancreas region × treatment), ^#^ *P* < 0.01 versus respective non-STZ injected group (interaction of pancreas region × treatment in panel (a) and main effect of treatment in panel (b).

Islet volume and mass were determined in control mice that consumed either LFD or HFD and mice that consumed either diet and received 3 injections of STZ. There was approximately 6.5 times greater volume of islets in the body region of the pancreas as compared with the head. While there was little difference in volume of islets in the head region of the pancreas among treatment groups, there was approximately a 50% reduction in islet volume in the body of the pancreas in response to 3 injections of STZ (Figure 9(a)). Consistently, total islet mass was reduced by about the same percentage in mice that received 3 injections of STZ than mice that consumed either the LFD or HFD.

## 4. Discussion

While peripheral insulin resistance is common during obesity and aging in mice and people, progression to T2D is largely due to insulin secretory dysfunction and significant apoptosis of functional *β* cells [13–17], leading to an inability to compensate for insulin resistance. As such, regeneration and preservation of functional *β*-cell mass becomes one of the fastest growing areas in diabetes research. While promotion of *β*-cell mass has been consistently shown as a promising strategy to treat diabetes [13, 14, 18–21], the majority of these studies have been carried out with obese diabetic (db/db) mice at relatively young ages. However, recent studies indicated that the capacity of *β*-cell regeneration is very limited in early middle-aged and older mice [22, 23], which raises an important question on the suitability of using young rodents to assess *β*-cell regeneration-based therapies for human T2D that occurs mostly in middle-aged and older individuals. In the present study, we generated a nongenetic mouse model of T2D using early middle-aged mice by feeding an HFD followed by multiple low doses of intraperitoneal STZ administration that did not cause diabetes in chow-fed mice. Like db/db mice, which is an expensive genetic obese diabetic mouse model, this mouse model shares the metabolic characteristics of human T2D, with peripheral insulin resistance and reduced *β*-cell mass and function [6, 24, 25]. However, unlike the db/db mice that develop overt and often severe diabetes at a very young age (4–8 wks) [3], a condition and age that are not closely pertinent to humans developing this disease, the diabetic model reported herein, which was generated with mice at 6 month of age, develops mild to moderate diabetes, and therefore may be more suitable as a T2D model for developing effective treatments and preventative strategies. In addition, the use of low doses of STZ in high fat-fed mice will also minimize the variability of diet-induced diabetes development and therefore provide better experimental controls for evaluating diabetic treatment strategies. 

As expected, mice that consumed the HFD were heavier than mice fed LFD and displayed slightly lower food intakes, presumably due to the greater caloric density of the high-fat diet. Injection of STZ slightly reduced food intake during the first few days but in general intakes fluctuated in a similar manner and remained relatively stable over the next 4 wk, with mice consuming more LFD as compared with mice that ate the HFD. Four or 5 injections of STZ reduced BW and the fat composition of mice that consumed HFD and reduced the weight of abdominal fat although it did not affect the weight of abdominal fat as a percentage of BW. In mice that consumed LFD however, there was a reduction in both fat composition and fat pad, both as an absolute weight and as proportion of BW, in response to 4 or 5 injections of STZ, which is also logical given that there was no effect of STZ on BW in those mice. The reductions in BW and body fat in severely diabetic mice (4 or 5 injections of STZ) occurred without a difference in food intake between these mice and mice from the other groups that consumed HFD. 

Blood lipid profile was influenced in this study by high-fat feeding and by STZ injection. Dyslipidemia, characterized by elevated blood cholesterol, triglycerides, phospholipids, and changes in lipoprotein composition, is a characteristic feature of T2D [26]. We observed a general trend that high-fat feeding and STZ injection resulted in elevated HDL and that total cholesterol and triglyceride levels were elevated in mice that ate the HFD and received 4 or 5 injections of STZ. Others have reported an influence of STZ injection on blood cholesterol, lipoprotein, and triglyceride concentrations [9, 27]. This could be due to elevated chylomicron and endogenous LDL formation as a result of consuming an HFD [9, 27]. While changes in these blood measurements tend to precede T2D, we did not observe significant changes in blood lipid profile in mice consuming the HFD without receiving STZ injections, suggesting that dyslipidemia in diabetic mice was modulated in part by the STZ. Others suggested that STZ leads to increased intestinal absorption and synthesis of cholesterol [9, 27]. STZ has a high affinity for the glucose transporter 2 (GLUT2) which is highly expressed in both the small intestine and pancreas, indicating that STZ could lead to changes in intestinal function [28].

While FBG levels vary among mouse strains and tend to be greater than those observed in humans, FBG at least 250 mg/dL is considered diabetic in mice [3]. The mice that consumed HFD and received either the vehicle or 1 injection of STZ displayed hyperinsulinemia without impaired FBG, characteristic of the metabolic state preceding T2D in humans whereby the individual is still able to maintain blood glucose homeostasis. While injection of 2 doses of STZ impaired glucose tolerance and greatly attenuated the HFD-induced hyperinsulinemia which is apparently required to counteract peripheral insulin resistance in these mice, HFD-fed animals treated with these STZ doses still had normal FBG, suggesting that this amount of STZ is still inadequate in destroying pancreatic *β*-cell mass to such an extent that animals become diabetic. 

Mice that consumed the HFD and received at least 3 injections of STZ maintained FBG levels greater than 250 mg/dL during the final 3 wk of the trial. However, mice that consumed the low-fat diet and received 5 injections of STZ displayed FBG levels that peaked at greater than 250 mg/dL during the 8th week of the trial. While mice that received 4 or 5 injections of STZ showed FBG levels greater than controls, regardless of diet, the HFD magnified the effect of the STZ, and mice that consumed HFD and received at least 3 injections of STZ displayed FBG levels that were greater than the respective mice that consumed LFD, indicating that feeding of an HFD and establishment of insulin resistance preceding diabetes enhances the action of the STZ. We observed reduced serum insulin levels in mice that received at least 2 injections of STZ and consumed HFD, as compared with the controls, and mice that consumed LFD and received 4 injections also showed reduced levels of circulating insulin, and those that received 5 injections had insulin levels that were not significantly lower although numerically lower than the controls. 

A glucose tolerance test was performed at 2 wk after STZ and insulin tolerance test at 3 wk after STZ. Impaired glucose tolerance was observed in mice that consumed HFD and received at least 2 injections of STZ. In mice that consumed HFD and received only 1 injection of STZ it is clear, judging from the data, that glucose tolerance is maintained in these animals by their ability to secrete sufficient quantities of insulin to compensate for impaired insulin resistance. In mice that consumed the LFD and received 4 or 5 injections of STZ, glucose tolerance was impaired, which is primarily due to destruction of pancreatic islets. However, feeding the HFD accentuated the differences. Evaluation of the glucose clearance rate showed that ability of mice to clear glucose from the blood after glucose levels reached peak values (30 min) was impaired in mice that consumed HFD and received at least 3 injections of STZ. Mice that consumed the LFD and received 5 injections of STZ showed a similar phenomenon. Because effects among mice that received 4 or 5 injections of STZ were so similar, regardless of diet, it appears that 4 and most definitely 5 injections of STZ at 40 mg/kg BW have an overly destructive effect in the pancreas. These data are in keeping with the idea that *β*-cell dysfunction preceded diabetes induced by STZ injection in mice that consumed the HFD.

Insulin sensitivity was influenced by the HFD alone and was more dramatically influenced by the combination of diet and STZ treatment. With the exception of mice that received 1 injection of STZ, all mice that consumed HFD displayed increased AUC as compared with mice that consumed LFD, demonstrating impairment in insulin sensitivity, independent of STZ treatment. The significant difference observed between the HFD and LFD group supports the procedure of feeding the HFD to induce impaired insulin sensitivity. Among the groups of mice that consumed HFD, those that received at least 3 injections of STZ displayed an even more pronounced impairment in insulin sensitivity, suggesting that at least 3 injections of STZ produce a true diabetic model. 

The evaluation of islet mass in this study provides a direct measure of the effect of STZ and the correlation to physiological parameters measured. There is a 60% reduction in relative pancreatic beta cell volume in obese T2D patients and 40% reduction in obese individuals with impaired FBG levels [29, 30]. We further evaluated islet volume and mass in mice that consumed HFD or LFD and received either the vehicle or 3 injections of STZ to determine if these doses of STZ are adequate to cause partial and significant *β*-cell destruction. Three injections of STZ, regardless of diet, resulted in approximately 50% destruction of islets, similar to the amount of lost *β* cells observed in human T2D. Apparently, destruction of this amount of *β* cells in HFD mice, but not in LFD mice, is adequate to cause diabetes. 

There are several explanations for the accentuated progression to diabetes after STZ injection in mice that consumed the HFD. The mice that consume the HFD gradually develop insulin resistance; we demonstrated that mice consuming the HFD showed impaired insulin sensitivity combined with greater insulin secretion, which is characteristic of many obese humans. The trigger is then the destruction of pancreatic *β* cells, leading to an inability for residual islets to secrete adequate amounts of insulin to compensate for insulin resistance. Indeed, the progression to T2D in humans with obesity is largely due to insulin secretory dysfunction and significant loss of functional *β* cells [31]. The chronic feeding of a high-fat diet may result in metabolic changes that alter the sensitivity of islets to toxic agents. Or, after ablation of cells by STZ treatment, remaining cells in mice fed the HFD may be dysfunctional as compared with the remaining cells in mice that consumed LFD, further reducing their ability to compensate for insulin resistance. The latter may be the case since histological analyses showed that islet mass was similar in mice that consumed either HFD or LFD and received 3 STZ injections, and serum insulin levels were different, suggesting that indeed, *β*-cell dysfunction preceded the full progression to diabetes induced by STZ injections. In conclusion, by feeding a high-fat diet and delivering consecutive low doses of STZ to older adult mice, we were able to optimize conditions for establishing a nongenetic and inexpensive mouse model that more closely mimics the metabolic profile that characterizes T2D in humans. This mouse model should be appropriate for future studies aimed at developing methods for T2D prevention and treatment.

## Figures and Tables

**Table 1:** Ingredient and chemical composition of diets.

	Standard chow		High-fat chow	
Ingredient	Amt (g)	Kcal	Amt (g)	kcal
Casein, 80 mesh	200	800	200	800
L-Cystine	3	12	3	12
Corn starch	315	1260	0	0
Maltodextrin 10	35	140	125	500
Sucrose	350	1400	68.8	275.2
Cellulose, BW200	50	0	50	0
Corn oil	25	225	25	225
Lard	20	180	245	2205
Mineral mix, S10026	10	0	10	0
Dicalcium phosphate	13	0	13	0
Calcium carbonate	5.5	0	5.5	0
Potassium citrate, 1H_2_0	16.5	0	16.5	0
Vitamin mix, V10001	10	40	10	40
Choline bitartrate	2	0	2	0
FD&C yellow dye ^#^5	0.05	0	0	0
FD&C red dye ^#^40	0	0	0.05	0

	Gm (%)	Kcal (%)	Gm (%)	Kcal (%)

Protein	19	20	26	20
Carbohydrate	67	70	26	20
Fat	4	10	35	60
Kcal/gram	3.8		5.2	

## References

[2] King H, Aubert RE, Herman WH (1998). Global burden of diabetes, 1995–2025: prevalence, numerical estimates, and projections. *Diabetes Care*.

[3] Clee SM, Attie AD (2007). The genetic landscape of type 2 diabetes in mice. *Endocrine Reviews*.

[4] Reuter TY (2007). Diet-induced models for obesity and type 2 diabetes. *Drug Discovery Today: Disease Models*.

[5] Leiter EH (2009). Selecting the “right“ mouse model for metabolic syndrome and type 2 diabetes research. *Methods in Molecular Biology*.

[6] Luo J, Quan J, Tsai J (1998). Nongenetic mouse models of non-insulin-dependent diabetes mellitus. *Metabolism*.

[7] Kahn BB, Flier JS (2000). Obesity and insulin resistance. *The Journal of Clinical Investigation*.

[8] Srinivasan K, Viswanad B, Asrat L, Kaul CL, Ramarao P (2005). Combination of high-fat diet-fed and low-dose streptozotocin-treated rat: a model for type 2 diabetes and pharmacological screening. *Pharmacological Research*.

[9] Islam MS, Choi H (2007). Nongenetic model of type 2 diabetes: a comparative study. *Pharmacology*.

[10] Fu Z, Zhang W, Zhen W (2010). Genistein induces pancreatic *β*-cell proliferation through activation of multiple signaling pathways and prevents insulin-deficient diabetes in mice. *Endocrinology*.

[11] Weibel ER (1972). The value of stereology in analysing structure and function of cells and organs. *Journal of Microscopy*.

[12] Kirk RE (1982). *Experimental Design: Procedures for the Behavioral Sciences*.

[13] Stoffers DA (2004). The development of beta-cell mass: recent progress and potential role of GLP-1. *Hormone and Metabolic Research*.

[14] Tourrel C, Bailbe D, Lacorne M, Meile MJ, Kergoat M, Portha B (2002). Persistent improvement of type 2 diabetes in the Goto-Kakizaki rat model by expansion of the *β*-cell mass during the prediabetic period with glucagon-like peptide-1 or exendin-4. *Diabetes*.

[15] Sakuraba H, Mizukami H, Yagihashi N, Wada R, Hanyu C, Yagihashi S (2002). Reduced beta-cell mass and expression of oxidative stress-related DNA damage in the islet of Japanese Type II diabetic patients. *Diabetologia*.

[16] Marchetti P, Del Guerra S, Marselli L (2004). Pancreatic islets from type 2 diabetic patients have functional defects and increased apoptosis that are ameliorated by metformin. *Journal of Clinical Endocrinology and Metabolism*.

[17] Cozar-Castellano I, Fiaschi-Taesch N, Bigatel TA (2006). Molecular control of cell cycle progression in the pancreatic *β*-cell. *Endocrine Reviews*.

[18] Sreenan S, Pick AJ, Levisetti M, Baldwin AC, Pugh W, Polonsky KS (1999). Increased *β*-cell proliferation and reduced mass before diabetes onset in the nonobese diabetic mouse. *Diabetes*.

[19] Suarez-Pinzon WL, Yan Y, Power R, Brand SJ, Rabinovitch A (2005). Combination therapy with epidermal growth factor and gastrin increases *β*-cell mass and reverses hyperglycemia in diabetic NOD mice. *Diabetes*.

[20] Wang Q, Brubaker P (2002). Glucagon-like peptide-1 treatment delays the onset of diabetes in 8 week-old db/db mice. *Diabetologia*.

[21] Rolin B, Larsen MO, Gotfredsen CF (2002). The long-acting GLP-1 derivative NN2211 ameliorates glycemia and increases beta-cell mass in diabetic mice. *American Journal of Physiology*.

[22] Rankin MM, Kushner JA (2009). Adaptive *β*-cell proliferation is severely restricted with advanced age. *Diabetes*.

[23] Tschen SI, Dhawan S, Gurlo T, Bhushan A (2009). Age-dependent decline in *β*-cell proliferation restricts the capacity of *β*-cell regeneration in mice. *Diabetes*.

[24] Strowski MZ, Li Z, Szalkowski D (2004). Small-molecule insulin mimetic reduces hyperglycemia and obesity in a nongenetic mouse model of type 2 diabetes. *Endocrinology*.

[25] Mu J, Woods J, Zhou YP (2006). Chronic inhibition of dipeptidyl peptidase-4 with a sitagliptin analog preserves pancreatic *β*-cell mass and function in a rodent model of type 2 diabetes. *Diabetes*.

[26] Goldberg IJ (2001). Clinical review 124: diabetic dyslipidemia—causes and consequences. *Journal of Clinical Endocrinology and Metabolism*.

[27] Mathe D (1995). Dyslipidemia and diabetes: animal models. *Diabete et Metabolisme*.

[28] Schnedl WJ, Ferber S, Johnson JH, Newgard CB (1994). STZ transport and cytotoxicity: specific enhancement in GLUT2-expressing cells. *Diabetes*.

[29] Matveyenko AV, Butler PC (2006). *β*-cell deficit due to increased apoptosis in the human islet amyloid polypeptide transgenic (HIP) rat recapitulates the metabolic defects present in type 2 diabetes. *Diabetes*.

[30] Wajchenberg BL (2007). *β*-cell failure in diabetes and preservation by clinical treatment. *Endocrine Reviews*.

[31] Donath MY, Ehses JA, Maedler K (2005). Mechanisms of *β*-cell death in type 2 diabetes. *Diabetes*.

